# Cost-benefit analysis of interventions for dementia: a scoping review

**DOI:** 10.1093/geroni/igaf084

**Published:** 2025-08-08

**Authors:** Tracy Comans, Tiet-Hanh Dao-Tran, Namal Balasooriya, Digby Simpson, Lee-Fay Low, Annica Barcenilla-Wong, Paola Vasquez, Junru Zhou, Kim-Huong Nguyen

**Affiliations:** Centre for Health Services Research, Faculty of Health, Medicine & Behavioural Sciences, University of Queensland, Brisbane, Queensland, Australia; National Ageing Research Institute Limited, Parkville, Victoria, Australia; Centre for Health Services Research, Faculty of Health, Medicine & Behavioural Sciences, University of Queensland, Brisbane, Queensland, Australia; School of Medicine and Dentistry, Griffith University, Gold Coast, Australia; Centre for Health Services Research, Faculty of Health, Medicine & Behavioural Sciences, University of Queensland, Brisbane, Queensland, Australia; School of Health Sciences, Faculty of Medicine and Health, University of Sydney, New South Wales, Australia; School of Health Sciences, Faculty of Medicine and Health, University of Sydney, New South Wales, Australia; Centre for Health Services Research, Faculty of Health, Medicine & Behavioural Sciences, University of Queensland, Brisbane, Queensland, Australia; Centre for Health Services Research, Faculty of Health, Medicine & Behavioural Sciences, University of Queensland, Brisbane, Queensland, Australia; Centre for Health Services Research, Faculty of Health, Medicine & Behavioural Sciences, University of Queensland, Brisbane, Queensland, Australia; Global Brain Health Institute, Trinity College Dublin, Dublin, Ireland

**Keywords:** Economic evaluation, Research methods, Healthcare policy, Evidence-based practice, Intervention

## Abstract

**Background and Objectives:**

Recently, cost-benefit analysis has been increasingly used to evaluate the value of interventions for dementia. This study aims to synthesize the methodology used in cost-benefit analysis (CBA) for dementia interventions.

**Research Design and Methods:**

We conducted a scoping review with comprehensive systematic searches for original peer-reviewed articles published from January 2010 to December 2023, and included the studies if they (1) performed a CBA of interventions for dementia, (2) described either cost or benefit items, and (3) performed quantitative data analysis on either costs or benefits. The review adhered to the PRISMA Extension for Scoping Reviews Checklist to write the report.

**Results:**

Of the 3415 articles found from the search, 15 were included in the review. Data analysis included the traditional CBA approach and its integration with the social return on investment approach. The set of cost and benefit items may vary depending on the intervention. Staff training, intervention supplies, building hire, and transportation were common cost items. Quality-adjusted life years (QALY), general practitioner visits, and emergency room visits were common benefit items. Cost data were often sourced from the study budget/assumptions. Benefit data were often sourced from the social value banks and literature. Market and shadow pricing were used for cost valuation. The value of statistical life was frequently used for benefit valuation.

**Discussion and Implications:**

This review synthesized data analysis methods, lists of cost and benefit items, data sources, and valuation methods used in the CBA of interventions for dementia. The findings provide helpful information for considering methodology in future CBA of interventions for dementia and similar interventions or conditions.

Transitional Significance: This review synthesized the methodological approach used for cost-benefit analysis (CBA). To be more specific, the review identified two data analysis methods, provided lists of cost and benefit items, and introduced data sources and valuation approaches for CBAs of interventions for dementia. Findings from this review provide helpful information for future users of CBA when considering methodology to conduct CBA interventions for dementia and similar interventions or conditions.

## Background and objectives

Dementia affects 55 million people globally.^1^ The number of people living with dementia will increase to 132 million by 2050.^2^ Dementia adversely affects the lives of individuals, carers, and the wider community.^2-9^ People living with dementia are less productive and require more care and medications.^5^ In some situations, they have more extended hospital stays and higher mortality rates.^4^ They may also experience stigma,^7^ depression, and anxiety.^6^ Costs related to dementia are projected to increase from 818 billion USD in 2015 to 2 trillion USD in 2050 worldwide.^2^

Many interventions have been developed and implemented to address dementia related challenges.^10^^,^^11^ To ensure that we allocate healthcare resources in the most optimal way, economic evaluations serve as an important tool.^12^^,^^13^ Economic evaluations consider the inputs (costs) and outputs (consequences) of an intervention and are classified based on how the consequences are valued and measured.^14^ Cost-effective analysis (CEA) and cost-utility analysis (CUA) value the consequence in natural units (ie, reduction in dementia cases).^14^ For CEA, the natural units are adjusted to quality-adjusted life years.^14^ This allows for the results to be presented as an incremental cost-effectiveness ratio, which can be considered as the price paid per outcome unit (ie, $/quality-adjusted life years [QALY] or $/dementia case avoided).^14^ In contrast, a cost-­benefit analysis (CBA) converts the health consequences into a monetary value to give a dollar value of the net benefit of an intervention.^14^

Cost-effective analysis and CUA are the most commonly applied approaches in healthcare settings.^15-19^ However, there has been a growing interest in using CBA in recent years, as CBA captures a wider range of outcomes, including societal and economic benefits beyond health.^20^ Cost-benefit analysis allows for comparison across diverse types of interventions, while CEA and CUA are more suited for comparing interventions that share similar health objectives. To date, the methodology approaches used for CBA of interventions for dementia seem heterogeneous. Understanding how CBA of interventions for dementia has been conducted would allow researchers to plan future CBA methodological approaches. This study aims to synthesize the data analysis approach, cost and benefit items, data sources, and valuation methods used in CBA of interventions for dementia. The study addresses the following research questions:

- What analysis approaches have been used in the CBA of interventions for dementia?
- What cost and benefit items have been measured in the CBA of interventions for dementia?
- Where have data on costs and benefits been collected?
- How have cost and benefit items been valued?

## Research designs and methods

### Designs

A scoping review following Arksey and O’Malley’s framework was conducted to address the study’s aim because it allows a comprehensive mapping of the existing diverse literature.^21^^,^^22^ Interventions for dementia include pharmacological and non-pharmacological interventions. Cost-benefit analysis of interventions for dementia may use various analysis approaches, cost and benefit items, data sources, and valuation methods. The study protocol was registered on Open Science Framework (OSF) Registries (https://doi.org/10.17605/OSF.IO/2XZDK).

### Study eligibility

Studies were included if they were original peer-reviewed articles published in full text between January 2010 and December 2023, performed a CBA of interventions for dementia, described either cost or benefit items, and performed quantitative data analysis on either costs or benefits. Studies were excluded if they performed other economic analyses other than CBA because the review's primary interest was benefits measured in monetary values. Studies that implied equal benefits or equal costs were excluded from the review. Commentaries, protocols, reviews, grey literature (conference abstracts, dissertations), and articles without full-text publications were excluded.

### Information sources

Searches were conducted in PubMed, MEDLINE, CINAHL, Embase, Scopus, PsycINFO, and EconLit electronic databases for all relevant articles published from January 2010 to December 2023. Search terms included CBA, dementia/Alzheimer’s disease (AD)/mild cognitive impairment (MCI), and intervention/program/framework/model/service. The search included AD and MCI because AD is the most common form of dementia, and MCI is conceptualized as prodromal dementia and often targeted for interventions for dementia during its early stages. A hand-search of reference lists of included articles supplemented the electronic database searches. The searches were completed on January 10, 2024 Details can be seen in Table S1).

### Study screening and selection

The articles found in database searches were imported into Covidence (https://www.covidence.org/) for reference management and the screening process. After removing duplicates, reviewers independently screened the titles and abstracts (N.B., D.S., J.Z.) and full texts (N.B., D.S., P.V.) for eligibility. A study was included if 2 reviewers independently agreed it met the selection criteria. Differences between reviewers were resolved in consensus discussions. If not, a third reviewer (H.D./K.H.N.) was included to decide on inclusion.

### Data extraction

The research team developed a template for data extraction. Charted data included author, year of publication, the country where the study was conducted, year of study, target population, type of dementia, stage of dementia, intervention, CBA approach, cost items, data sources for costs, cost valuation methods, benefit items, measurement of benefits, data sources for benefits, and benefits valuation approach. Two reviewers (J.Z. and H.D.) independently extracted data on the study characteristics. Two other reviewers (D.S. and N.B.) extracted data on CBA. After collecting data extraction results, any conflicts were resolved by discussions with a third reviewer (K.H.N.).

### Quality assessment of the included studies

Two reviewers (P.V. and K.H.N.) independently appraised the quality of the included studies using 28 items of the Consolidated Health Economic Evaluation Reporting Standards (CHEERS) 2022 checklist.^23^ Although the CHEERS 2022 Checklist was designed to appraise the reporting quality of CEA studies, we believe it is (currently) the closest and most appropriate checklist available to appraise reporting quality for CBA studies, given that there is a quality checklist designed for CBA studies.

### Data analysis and presentation of the results

The PRISMA Extension for Scoping Reviews Checklist ­(PRISMA-ScR) guided the report writing.^24^ Cost items were classified into labor, interventions and medical services, materials, rents, and other expenses (such as transportation, accommodations, meals, and advertisements). Benefit items were classified into death, quality of life, mental health, psychosocial function, resource use, benefits for caregivers and friends, healthcare professionals, and the wider community/society. The classification process and outcomes were conducted by one researcher (H.D.) and then reviewed by the team for consensus agreement. The final findings were presented in figures, tables, and narrative texts.

## Results

### Search and screening outcomes

**Figure 1.**
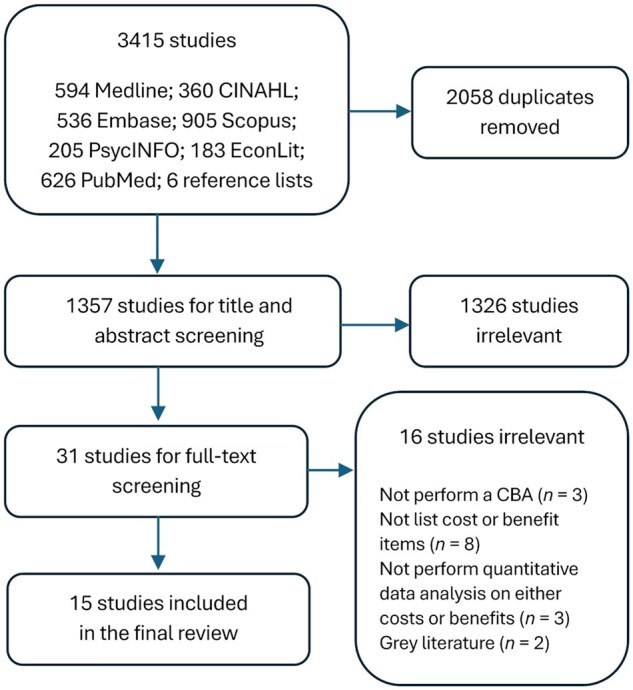
The preferred reporting items for scoping reviews (PRISMA-ScR) flowchart.

The database searches found 3415 articles, 2058 of which were duplicates and were removed. After title and abstract screening, 31 studies were included for full-text review. The full-text review further excluded 16 studies because they (1) did not perform a CBA (*n* = 3), (2) did not list cost or benefit items (*n* = 8), (3) did not perform quantitative data analysis on either costs or benefits (*n* = 3); or (4) were gray literature (*n* = 2). No additional articles were found outside the electronic database searches. The review included 15 peer-reviewed articles (see Figure 1).

### Study characteristics

As seen in Table 1, all included studies were published between 2018 and 2023, with 6 published in 2022.^25^-30 Brent published 6 studies.^25^^,^^26^^,^^31-33^ Where reported, most studies were conducted in high-income countries (United States [*n* = 6],^25-27^^,^^31-33^ Canada [*n* = 2],^28^^,^^34^ Australia [*n* = 2],^35^^,^^36^ United Kingdom [*n* = 3],^29^^,^^37^^,^^38^ Japan [*n* = 1],^39^ not specified [*n* = 1])^30^ Six of 15 studies did not state when the study took place.^20^^,^^25^^,^^30-33^ When reported, studies were conducted with data from 2012 to 2020 and were often conducted over 2 to 3 years. Studies predominantly had interventions targeting either people living with dementia (*n =* 8) or the dementia dyads (people living with dementia and their informal caregivers (*n =* 5).^27^^,^^29^^,^^30^^,^^35^^,^^38^ One study targeted people with memory concerns,^28^ and one targeted children.^36^ Of those that included people living with dementia (*n =* 13), 11 did not specify the type of dementia; one evaluated an intervention for people with AD,^31^ and one evaluated an intervention for people with mixed dementia diagnoses.^32^ Seven studies did not report the dementia stage,^26^^,^^28^^,^^30^^,^^34^^,^^35^^,^^37^^,^^38^ 5 included people living with dementia of mixed stages,^25^^,^^31-33^^,^^39^ and 1 focused on early dementia.^29^ In addition to pharmacological interventions,^31^ non-pharmacological interventions that enhanced cognition,^28^^,^^37^^,^^39^ sensory functions,^32^^,^^33^ physical activity,^29^ peer support,^38^ health services,^34^ public awareness,^36^ and laws^30^ were evaluated using CBA.

**Table 1.** Study characteristics.

ID	Lead author	Country	Publication year	Study period	Targeted population	Dementia type	Dementia stage	Interventions
**1**	Brent	USA	2019	2005-2015	People living with dementia	Mixed^a^	Mixed	Hearing aids
**2**	Brent	USA	2020	2005-2015	People living with dementia	NR	Mixed	Corrective lenses
**3**	Brent	USA	2022	2005-2015	People living with dementia	NR	Mixed	Avoiding nursing homes
**4**	Brent	USA	2022	NR	People living with dementia	NR	NR	Specialized unit for the prevention and treatment of elder abuse
**5**	Brent	NR	2022	NR	People living with dementia + informal caregivers	NR	NR	Law changes: Laws for people living with dementia participating in cognitive rehabilitation programs (including the Tailored Activity Program)
**6**	Brent	USA	2023	2005-2015	People living with dementia	AD	Mixed	FDA-approved medications
**7**	Hartfiel	UK	2022	2016-2018	People living with dementia + informal caregivers	NR	Mild	PrAISED programme, an individually tailored, home-based supervised exercise program for people living with dementia
**8**	Jones	England + Wales	2020	2014-2015	People living with dementia	NR	NR	Visual arts program
**9**	Willis	UK	2018	2012-2014	People living with dementia + informal caregivers	NR	NR	Peer support groups
**10**	Rahja	Australia	2020	2016-2019	People living with dementia + informal caregivers	NR	NR	Evidence-based dementia care program
**11**	Sado	Japan	2020	2015-2016	People living with dementia	NR	Mixed	Learning therapy
**12**	Baker	Canada	2022	2012-2020	Older adults with memory concerns	NA	NA	Group memory intervention
**13**	Pizzi	USA	2022	2015-2019	People living with dementia + informal caregivers	NR	NR	Care of people living with dementia in their environments program
**14**	Smith	Australia	2020	2018	Children	NA	NA	Education to a second party does not directly address the person affected by dementia.
**15**	Thanh	Canada	2020	2017-2019	People living with dementia	NR	NR	Primary Health Care Integrated Geriatric Services

a Mixed: includes different stages of dementia rather than one specific stage.

Abbreviations: AD, Alzheimer’s disease; CBA, cost-benefit analysis; CEA, cost-effectiveness analysis; NA, not applicable; NR, not reported; UK, United Kingdom; USA, United States of America.

### Data analysis approaches

Two CBA approaches were used across 15 papers, including traditional CBA analysis^25-28^^,^^30-33^^,^^35^^,^^36^^,^^39^ and CBA incorporating social return on investment (SROI).^29^^,^^34^^,^^37^^,^^38^ The traditional CBA systematically values the gains and losses for individuals in monetary terms.^40^ The CBA incorporates SROI, quantifying costs and benefits in monetary terms; however, it also considers benefits to broader groups such as family, society, and the wider community.^29^^,^^34^^,^^37^^,^^38^

### Cost items, data sources, and valuation approaches

As seen in Table 2, across 15 included studies, 25 cost items were used, including labor (11 items), interventions and medical services (5 items), materials (4 items), rents (1 item), and other expenses (4 items) (such as transportation, accommodations, meals, and advertisements). Different sets of cost items were used for different interventions. Different interventions could classify one cost item as a direct or indirect cost. The common cost items across studies were staff training (*n =* 6), intervention supplies (*n =* 6), building hire (*n =* 5), and transportation costs (*n =* 6). Market pricing (actual [budget]) and shadow pricing (estimated or assumed cost) were applied for cost valuation. The cost estimates were often sourced from the literature.

**Table 2.** Cost items, sources of data, and validation methods for costs.

Cost domains and items	ID	Source of data	Valuation methods
**Labor**			
**1. Staff educators for training**	7	Study assumption	Market price (direct cost)
	10	Study budget	Market price (direct cost)
	11	Study assumption using National wage survey	Market price (direct cost)
	12	Study assumption	Market price (direct cost)
	13	Study budget	Market price (direct cost)
	14	Study assumption using the Australian Bureau of statistics	Shadow price (indirect cost)
**2. Caregiver time**	5	Study survey using Minimum wage	Market price (direct cost)
**3. Volunteers’ unpaid time**	9	Study assumption using Minimum wage	Shadow price (indirect cost)
**4. Lawyer time (time spent on persecution)**	4	Sourced from literature	Market price/average wage (indirect cost)
**5. PT/OT time**	5	Study budget	Market price/average wage (direct cost)
	7	Study assumption using NHS health worker pay scale	Market price/average wage (direct cost)
	10	Study assumption using Queensland health worker pay scale	Shadow price (indirect cost)
	13	Study budget	Market price/average wage (direct cost)
**6. Physician time**	6	NACC data set using Medicare item code	Market price/average wage (direct cost)
**7. Registered social worker time**	7	Study assumption using NHS health worker pay scale	Market price/average wage (direct cost)
**8. Home care worker time**	8	Study assumption using literature	Market price/average wage (direct cost)
**9. Participant time**	8	Study assumption using minimum wage	Shadow price (indirect cost)
**10. Nursing time**	10	Study assumption using Queensland health worker pay scale	Shadow price (indirect cost)
**11. Administrative assistant time**	12	Study assumption	Market price (direct cost)
***Interventions and medical services***			
**1. Interventions (screening for program eligibility, programs and workshops, setup and management)**	14	Study budget	Market price (direct cost)
	15	Study budget	Market price (direct cost)
**2. Eye examination**	2	Study assumption using literature	Market price (direct cost)
**3. Clinical monitoring (drug reaction)**	6	NACC data set using Medicare item code using literature	Market price (direct cost)
**4. Home care**	6	NACC data set using literature	Shadow price (indirect cost)
**5. Post-fitting audiological rehabilitation support services**	1	Study assumption using literature	Market price (direct cost)
***Materials***			
**1. Hearing aids**	1	Sourced from literature	Market price (direct cost)
**2. Eyeglasses**	2	Study assumption using literature	Market price (direct cost)
**3. Intervention supplies, including stationery and prints, and exercise equipment**	5	Study budget	Market price (direct cost)
	7	Study assumption	Market price (direct cost)
	10	Study budget	Market price (direct cost)
	11	Study budget	Market price (direct cost)
	12	Study assumption	Market price (direct cost)
	13	Study budget	Market price (direct cost)
**4. Medication**	6	NACC data set using US Centers for Medicare and Medicaid Services	Market price (direct cost)
***Rents***			
**1. Building hire (venue hire, room rental)**	7	Study assumption	Market price (direct cost)
	8	Study budget	Shadow price (indirect cost)
	9	Study assumption using cost of similar renting	Shadow price (indirect cost)
	10	Study assumption	Shadow price (indirect cost)
	12	Study assumption	Market price (direct cost)
***Other expenses***			
**1. Transportation**	5	Study budget	Market price (direct cost)
	7	Study assumption	Shadow price (indirect cost)
	10	Study budget	Market price (direct cost)
	13	Study budget	Market price (direct cost)
	14	Study budget	Market price (direct cost)
	15	Study budget	Market price (direct cost)
**2. Accommodation**	10	Study budget	Market price (direct cost)
	15	Study budget	Market price (direct cost)
**3. Meals**	7	Study assumption	Market price (direct cost)
	10	Study budget	Market price (direct cost)
	12	Study assumption	Market price (direct cost)
	13	Study budget	Market price (direct cost)
**4. Advertisement of program**	12	Study assumption	Market price (direct cost)

### Benefit items, data sources, and valuation approaches

As seen in Table S2, a total of 49 benefit items were used, including death (2 items), quality of life (1 item), mental health (2 items), psychosocial (8 items), function (3 items), resource use (16 items), benefits for caregivers and friends (6 items), benefits for healthcare professionals (4 items), and benefits for the wider community/society (7 items). Different interventions have different sets of benefit items. The QALY (*n =* 4), general practitioner visits (*n =* 4), and emergency visits (*n =* 4) are considered common benefit items. There was a large variation in how benefits were measured. These measurement outcomes were converted into monetary value using various data sources and valuation methods. Social value banks and literature were often the data sources for benefits, and the value of statistical life was commonly used for benefit valuation. Long-term and broad-ranged benefit items were not always included or measured due to the restricted observation time frame or limited measurement method.

### Quality appraisal of included studies

The quality assessment of the included studies using the CHEERS 2022 checklist (see Table 3) found that 6 studies met more than 70% of the required reporting criteria.^25-27^^,^^32^^,^^33^^,^^36^ Brent published 4 of these 6 studies. Five high-quality studies were conducted in the United States.^25-27^^,^^32^^,^^33^ Most studies provided thorough reports across several key areas, including the title, abstract, background, objectives, analytical perspectives, and the PICO (study population, intervention, comparator, and outcome selection) framework. The studies often offered detailed accounts of outcome and cost measurement, main findings, study limitations, generalizability, funding sources, and declarations of conflicts of interest. Multiple studies failed to report crucial elements such as the health economic analysis plan, time horizon for the analysis, discount rate, rationale and description of the chosen modeling approach, analytical assumptions, consideration of distributional effects, uncertainty in parameter estimates, and strategies for engaging patients and other stakeholders affected by the study.

**Table 3.** Quality assessment of the included studies using the CHEERS checklist

Criteria	ID														
	[1]^32^	[2]^33^	[3]^25^	[4]^26^	[5]^30^	[6]^31^	[7]^29^	[8]^37^	[9]^38^	[10]^35^	[11]^39^	[12]^28^	[13]^27^	[14]^36^	[15]^34^
**1. Title**	1	1	1	1	1	0	1	1	1	1	0.5	1	1	0	1
**2. Abstract**	1	1	1	1	1	1	1	1	1	1	0.5	0	1	0.5	1
**3. Background, objectives**	1	1	1	1	1	1	1	1	1	1	1	1	1	1	1
**4. Health economic analysis plan**	1	1	0	0	0	1	1	0	1	0.5	0	0	0	0	0
**5. Study population**	1	1	1	1	1	1	1	1	1	1	1	0	1	1	1
**6. Setting**	1	1	1	1	1	1	1	1	1	1	1	1	1	1	1
**7. Comparators**	1	1	1	1	1	1	1	1	1	1	1	0.5	1	1	1
**8. Perspective**	1	1	1	1	1	1	1	1	1	1	0.5	0.5	1	0	1
**9. Time horizon**	1	1	1	0	0	1	0	0	0	0	0	1	1	0	1
**10. Discount rate**	1	1	0	0	0	1	0	0	0	1	0	0	0	0	0
**11. Selection of outcomes**	1	1	1	1	1	1	1	1	1	1	1	0.5	1	1	1
**12. Measurement of outcomes**	1	1	1	1	1	1	1	1	1	1	1	0	1	1	1
**13. Valuation of outcomes**	1	1	1	1	1	1	1	1	1	1	1	0	1	0.5	0
**14. Measurement and valuation of resources and costs**	1	1	1	1	1	1	1	1	1	1	1	1	1	0.5	0
**15. Currency, price date, and conversion**	1	1	1	1	1	1	0	0	0	1	1	1	1	1	0.5
**16. Rationale, description of the model**	1	1	1	1	1	1	0	0	0	0.5	0.5	0	0	0.5	0
**17. Analytics and assumptions**	1	1	1	1	1	1	0	0	0	0.5	0.5	0	0	0	0.5
**18. Characterizing heterogeneity**	1	1	1	1	0	1	0	0	0	0.5	0	0	0	0	0
**19. Characterizing distributional effects**	1	1	1	1	0	1	0	0	0	0.5	0	0	0	0	0
**20. Characterizing uncertainty**	1	1	1	1	0	1	0	0	0	1	0.5	0.5	1	0	0.5
**21. Approach to engage with patients and others affected by the study**	0	1	0	0	0	0	1	1	1	0	0	0	0	0	0
**22. Study parameters**	1	1	1	1	1	1	1	1	1	1	0.5	0	1	1	1
**23. Summary of main results**	1	1	1	1	1	1	1	1	1	1	1	0.5	1	1	1
**24. Effect of uncertainty**	1	1	1	1	0	1	0	0	0	0.5	0	0	0	0	1
**25. Effect of engagement with patients and others affected by the study**	1	1	1	1	1	1	1	1	1	0.5	0	0.5	0.5	0	0.5
**26. Study findings, limitations, generalizability, and current knowledge**	1	1	1	1	1	1	1	1	1	1	1	0.5	1	0.5	1
**27. Source of funding**	1	1	0	0	0	1	1	1	1	1	1	1	1	1	0
**28. Conflicts of interest**	1	1	0	0	0	1	1	1	1	1	1	1	1	1	0
**% of CHEERS criteria met**	96.4%	100.0%	82.1%	78.6%	64.3%	92.9%	67.9%	64.3%	67.9%	80.4%	58.9%	41.1%	69.6%	48.2%	57.1%

A score of 1 was assigned for quality details, a score of 0.5 for limited detail, and a score of 0 for missing details. ID: [1] Brent (2019), [2] Brent (2020), [3] Brent (2022), [4] Brent (2022), [5] Brent (2022), [6] Brent (2024), [7] Hartfiel (2022), [8] Jones (2020), [9] Willis (2018), [10] Rahja (2020), [11] Sado (2020), [12] Baker (2022), [13] Pizzi (2022), [14] Smith (2020), [15] Thanh (2020).

## Discussion and implications

This scoping review included 15 studies and synthesized the use of CBA of interventions for dementia. The review found 2 data analysis approaches, 25 cost items, and 49 benefit items, used across CBA interventions for dementia. Different sets of cost and benefit items for each intervention were identified. Four cost items (staff training, intervention supplies, building hire, and transportation) and 3 benefit items (QALY, general practitioner visit, emergency room visit) were commonly used across different interventions. Cost data were often sourced from the study budget/assumptions. Both market pricing and shadow pricing were used for cost valuation. There was a large variation in how benefits were measured; sometimes, they were only measured but not assigned any value. The social value of banks and literature were common sources for benefits, and the value of statistical life was often used for benefit valuation. Future users of CBA should be mindful of the heterogeneity in the CBA methodology approach, especially for benefits, and select a suitable choice for their CBA.

Unlike acute health conditions, where interventions are more likely to target patients and treatments, interventions for dementia may target people living with dementia, their caregivers and families, healthcare professionals, and the wider community/society—for example, public awareness^36^ and stigma reduction, and law enforcement.^30^ The interventions for dementia have a broader scope of impact. Therefore, the interest in using CBA is increasing.^20^ The results from the CBA can provide policymakers with evidence about the tradeoff between benefits and costs borne by different stakeholders.^20^

It is noted that CBAs have only recently been used in the medical and healthcare evaluation literature on dementia. This marks a shift toward broader economic assessments that capture the impacts of dementia interventions beyond health and the dementia dyads. This starkly contrasts with the rich literature on CEA of interventions for dementia.^13^^,^^41-45^ Future users should be aware of the availability of different economic analyses and compare and contrast CBA with other methods to select the more suitable economic valuation approach, depending on how the outcomes are valued, the scope of measurement, and the implications to generate valuable evidence supporting informed policy decisions on investments in dementia care.

Cost items were more easily identified than benefits because they are often directly related to interventions. The review highlighted the variation of included cost items across interventions, identified the common cost items across interventions, and provided a synthesized list of 25 cost items for users to consider other relevant items for a specific intervention that may have been missed, not identified, and evaluated in the existing CBA. Caregivers’ productivity loss may also be considered a cost item to acknowledge the caregiver’s burden.^46^ The information generated for this review should be used for consideration rather than being a “gold standard” to determine which cost items to value.

Market pricing is a valuation approach to the value of a good or service as determined by the price revealed in market transactions. It is based on actual transactions and reflects a specific market's supply and demand dynamics. Shadow pricing is a valuation approach that assigns a value to goods, services, or resources not traded in the market or when market prices do not reflect their actual social or economic value. Using market pricing for cost valuation can provide a more precise valuation than shadow pricing. Yet, costs may differ in different settings and over time. However, as several existing studies did not report the year of study, it is difficult to compare the monetary values reported across studies, limiting the translation of those findings into other contexts. As per the CHEERS 2022 checklist, including a description of the setting and time when reporting cost valuation is crucial for accurately interpreting the study findings.

The review also demonstrated a significant variation in benefit items and how benefits were measured. The review also revealed that sometimes benefits were only mentioned but not measured or assigned any value. The identified common benefit items and the list of 49 items used across different interventions allow users to consider relevant items for their CBA. A larger number of benefit items and various measurement approaches reflect 2 phenomena. First, it mirrors the heterogeneity of interventions evaluated in this literature. Each intervention has a different impact mechanism, resulting in various stakeholder benefits. Second, the data and valuation methods needed to capture the benefits might not yet exist or be appropriately developed for the dementia space, leading to the practice of borrowing from other literature and sources. Therefore, precautions should be taken when interpreting and translating evidence generated from CBA. This presents an excellent opportunity for future research to improve the data collection and evaluation methodologies so that the CBA results are better suited for the purpose and can stand methodological scrutiny. A Lancet report published in 2020 also mentioned some other cost and benefit items from the intervention related to dementia, which can also be considered in future CBA.^47^ As a developing topic, the CBA method is going through adjustments and adaptations to suit the evaluation purposes, especially to answer the decision questions by funders and governments.

To date, most CBAs of interventions for dementia have been conducted in developed countries, notably the United States,^25-27^^,^^31-33^ where research and development in dementia interventions have received the most investment. Even though Brent discussed the implications for low and middle-income countries (LMICs),^25^^,^^26^^,^^30^^,^^32^^,^^33^ it is important to note that evidence translation remains hypothetical. Globally, the dementia burden falls more heavily on low and middle-income countries.^1^ This geographic skew limits the generalizability of findings to LMICs, where the health and care systems differ markedly in structure (health financing included) and service availability. Public health systems in these countries are often under-resourced, while private sector coverage is minimal, covering mostly the top-income tier of the population. This leads to substantial variation in cost structures: cost-effective interventions in HICs may be prohibitively expensive in LMICs due to infrastructure requirements (eg, diagnostic imaging, specialist labor, pharmacological supply chains). Even when cost components are conceptually applicable, their unit costs and feasibility differ dramatically.

The role of informal care in LMICs substantially alters the economic analysis, which was often referred to as “spillover effect” in the HIC studies, due to the underdeveloped formal systems for long-term care services (residential facilities or dementia-specific day programmes), thus, a shortage of formal care. Consequently, family members and community networks serve as primary carers, often at great personal cost: time, finance/income, and health. This type of cost shifting, from the public to private households and communities, makes the valuation of informal care a critical component of cost-benefit analyses in these settings.

Lastly, the effectiveness of an intervention or programme might not be transferable from HICs to LMICs. Implementation quality, population characteristics, and cultural adaptation all influence outcomes; for instance, an intervention proven beneficial in a highly resourced, specialist-led system may require substantial modification, resulting in different effects in a low-resource, community-based context, because it might evolve into a different intervention. These variations demand caution in applying high-income evidence to LMIC policy decisions.

### Strengths and limitations

This review conducted comprehensive systematic searches, adhered to Arksey and O’Malley’s framework,^21^^,^^22^ had at least 2 authors independently perform study screening and data extraction for each study, and followed the PRISMA-ScR Checklist^24^ to write a report demonstrating a robust methodology. In addition, some standards in the CHEERS Checklist may not apply to CBA studies due to methodological variations. Compared to the initial CHEERS Checklist, the CHEERS 2022 Checklist added new aspects such as a health economic analysis plan, model sharing, and the increasing involvement of stakeholders and engagement with communities, patients, and the public in health research.^23^ These aspects are evolving and becoming critical in CEA practice and have not yet been frequently visible in other literature, such as CBA. We suspect that the overall reporting quality is lower than that of the CEA/CUA literature. For instance, several studies failed to meet the reporting criteria.

### Implications for future practice, policy, and research

Interventions for dementia may influence not only people living with dementia but also their caregivers, families, healthcare professionals, and the wider community/society. These interventions may have short-term and long-term impacts on many outside health domains. In situations where multiple sectors and impact domains with a broad society and stakeholders are involved, it might be beneficial to consider CBA as an alternative to CEA. Methodologically, it is possible to conduct a parallel evaluation of CEA and CBA, which can serve as a comparative analysis and contrast the economic evidence generated by both methods. This practice will provide additional information to support a broader debate on efficient and equitable investments in dementia care.

Like other economic evaluations, CBA is often conducted at the end of the intervention. However, for a high-quality CBA, the methodology should be thoroughly planned at the beginning of the project with justification for its appropriateness. The selected method of economic evaluation should be justified. Planning of cost and benefit items, data sources, valuation methods, and the proposed adjustment and sensitivity analyses plan is highly recommended. In addition, the study year, the type, and stage of dementia should be reported. The information is essential for readers to justify the study’s findings and be more confident in the evidence from the published research.

## Conclusion

This scoping review highlights the increasing use of CBA as an economic evaluation method to establish evidence of the value of interventions for dementia. While CBA offers valuable insights into the distribution of costs and benefits across individuals, caregivers, and broader society, reporting limitations and heterogeneity in methodological approach, especially for benefits across existing CBA of interventions for dementia, should be considered. The review outlined methodological choices for CBA of interventions for dementia and similar interventions for similar conditions in the future.

## Supplementary Material

igaf084_Supplementary_Data

## Data Availability

All available data are presented in Tables or supplementary files.
