# Supplementary material for: Cost-benefit analysis of interventions for dementia: a scoping review
Source: Innov Aging. 2025 Aug 8;9(8):igaf084. doi: 10.1093/geroni/igaf084 (PMC12448809; doi:10.1093/geroni/igaf084)
Supplement: igaf084_Supplementary_Data [file igaf084_supplementary_data.docx]

***Innovation in Aging* Supplementary Material:** **Comans, Dao-Tran, Balasooriya, Simpson, Low, Barcenilla-Wong, Vasquez, Zhou & Nguyen. Cost-Benefit Analysis of Interventions for Dementia: A Scoping Review**

Supplementary Table 1. Search terms and results

| **Databases** | **Search terms** | **Results** |
| --- | --- | --- |
| **PubMed** | ("Dementia"[Mesh] OR "Alzheimer Disease"[Mesh] OR "Cognitive Dysfunction"[Mesh] OR “dementia”[tiab] OR “alzheimer*”[tiab] OR “cognitive impairment*”[tiab] OR “cognitive dysfunction*”[tiab] OR “cognitive disorder*”[tiab] OR “cognitive decline*”[tiab])  **AND**  (“Intervention*”[tiab] OR “Program*”[tiab] OR “Framework*”[tiab] OR “Model*”[tiab] OR “Service*”[tiab] OR “screening” [tiab] OR “prevention*”[tiab] OR “treatment*”[tiab])  **AND**  ("Cost-Benefit Analysis"[Mesh] OR “cost benefit”[tiab] OR “cost benefits”[tiab] OR “costs benefit”[tiab] OR “costs benefits”[tiab] OR “cost-benefit”[tiab] OR “cost-benefits”[tiab] OR “costs-benefit”[tiab] OR “costs-benefits”[tiab] OR “economic evaluation*”[tiab])  **AND** (2010:2023[dp]) **AND** (eng[la] OR und[la]) | 626 |
| **Medline (EBSCOhost)** | (MH "Dementia+" OR MH "Alzheimer Disease" OR MH "Cognitive Dysfunction+" OR TI(“dementia” OR “alzheimer*” OR “cognitive impairment*” OR “cognitive dysfunction*” OR “cognitive disorder*” OR “cognitive decline*”) OR AB(“dementia” OR “alzheimer*” OR “cognitive impairment*” OR “cognitive dysfunction*” OR “cognitive disorder*” OR “cognitive decline*”))  **AND**  (TI(“Intervention*” OR “Program*” OR “Framework*” OR “Model*” OR “Service*” OR “screening”  OR “prevention*” OR “treatment*”) OR AB(“Intervention*” OR “Program*” OR “Framework*” OR “Model*” OR “Service*” OR “screening”  OR “prevention*” OR “treatment*”))  **AND**  (MH "Cost-Benefit Analysis" OR TI(“cost benefit” OR “cost benefits” OR “costs benefit” OR “costs benefits” OR “cost-benefit” OR “cost-benefits” OR “costs-benefit” OR “costs-benefits” OR “economic evaluation*”) OR AB(“cost benefit” OR “cost benefits” OR “costs benefit” OR “costs benefits” OR “cost-benefit” OR “cost-benefits” OR “costs-benefit” OR “costs-benefits” OR “economic evaluation*”))  **AND** (DT 20100101-20231231) **AND** (LA English) | 594 |
| **Embase (Elsevier)** | ('dementia'/exp OR 'Alzheimer disease'/exp OR 'cognitive defect'/exp OR “dementia”:ti,ab OR “alzheimer*”:ti,ab OR “cognitive impairment*”:ti,ab OR “cognitive dysfunction*”:ti,ab OR “cognitive disorder*”:ti,ab OR “cognitive decline*”:ti,ab)  **AND**  (“Intervention*”:ti,ab OR “Program*”:ti,ab OR “Framework*”:ti,ab OR “Model*”:ti,ab OR “Service*”:ti,ab OR “screening”:ti,ab OR “prevention*”:ti,ab OR “treatment*”:ti,ab)  **AND**  ('cost benefit analysis'/exp OR “cost benefit”:ti,ab OR “cost benefits”:ti,ab OR “costs benefit”:ti,ab OR “costs benefits”:ti,ab OR “cost-benefit”:ti,ab OR “cost-benefits”:ti,ab OR “costs-benefit”:ti,ab OR “costs-benefits”:ti,ab OR “economic evaluation*”:ti,ab)  **AND** [2010-2023]/py AND [english]/lim **AND** ([article]/lim OR [article in press]/lim OR [review]/lim) | 536 |
| **CINAHL Complete (EBSCOhost)** | (MH "Dementia+" OR MH "Alzheimer's Disease" OR MH "Cognition Disorders+" OR TI(“dementia” OR “alzheimer*” OR “cognitive impairment*” OR “cognitive dysfunction*” OR “cognitive disorder*” OR “cognitive decline*”) OR AB(“dementia” OR “alzheimer*” OR “cognitive impairment*” OR “cognitive dysfunction*” OR “cognitive disorder*” OR “cognitive decline*”))  **AND**  (TI(“Intervention*” OR “Program*” OR “Framework*” OR “Model*” OR “Service*” OR “screening”  OR “prevention*” OR “treatment*”) OR AB(“Intervention*” OR “Program*” OR “Framework*” OR “Model*” OR “Service*” OR “screening”  OR “prevention*” OR “treatment*”))  **AND**  (MH "Cost Benefit Analysis" OR TI(“cost benefit” OR “cost benefits” OR “costs benefit” OR “costs benefits” OR “cost-benefit” OR “cost-benefits” OR “costs-benefit” OR “costs-benefits” OR “economic evaluation*”) OR AB(“cost benefit” OR “cost benefits” OR “costs benefit” OR “costs benefits” OR “cost-benefit” OR “cost-benefits” OR “costs-benefit” OR “costs-benefits” OR “economic evaluation*”))  **AND** (PY 2010-2023) **AND** (LA English) | 360 |
| **Scopus, Advanced Search (Elsevier)** | (TITLE-ABS-KEY(“dementia” OR “alzheimer*” OR “cognitive impairment*” OR “cognitive dysfunction*” OR “cognitive disorder*” OR “cognitive decline*”))  **AND**  (TITLE-ABS-KEY(“Intervention*” OR “Program*” OR “Framework*” OR “Model*” OR “Service*” OR “screening”  OR “prevention*” OR “treatment*”))  **AND**  (TITLE-ABS-KEY(“cost benefit” OR “cost benefits” OR “costs benefit” OR “costs benefits” OR “cost-benefit” OR “cost-benefits” OR “costs-benefit” OR “costs-benefits” OR “economic evaluation*”))  **AND** PUBYEAR > 2009 AND PUBYEAR < 2024 **AND** ( LIMIT-TO ( LANGUAGE,"English" ) ) | 904 |
| **PsycINFO (EBSCOhost)** | (DE "Dementia" OR DE "AIDS Dementia Complex" OR DE "Alzheimer's Disease" OR DE "Dementia with Lewy Bodies" OR DE "Frontotemporal Lobar Degeneration" OR DE "Presenile Dementia" OR DE "Pseudodementia" OR DE "Senile Dementia" OR DE "Vascular Dementia" OR DE "Alzheimer's Disease" OR DE "Cognitive Impairment" OR DE "Mild Cognitive Impairment" OR TI(“dementia” OR “alzheimer*” OR “cognitive impairment*” OR “cognitive dysfunction*” OR “cognitive disorder*” OR “cognitive decline*”) OR AB(“dementia” OR “alzheimer*” OR “cognitive impairment*” OR “cognitive dysfunction*” OR “cognitive disorder*” OR “cognitive decline*”))  **AND**  (TI(“Intervention*” OR “Program*” OR “Framework*” OR “Model*” OR “Service*” OR “screening”  OR “prevention*” OR “treatment*”) OR AB(“Intervention*” OR “Program*” OR “Framework*” OR “Model*” OR “Service*” OR “screening”  OR “prevention*” OR “treatment*”))  **AND**  (DE "Costs and Cost Analysis" OR TI(“cost benefit” OR “cost benefits” OR “costs benefit” OR “costs benefits” OR “cost-benefit” OR “cost-benefits” OR “costs-benefit” OR “costs-benefits” OR “economic evaluation*”) OR AB(“cost benefit” OR “cost benefits” OR “costs benefit” OR “costs benefits” OR “cost-benefit” OR “cost-benefits” OR “costs-benefit” OR “costs-benefits” OR “economic evaluation*”))  **AND** (PY 2010-2023) **AND** (LA English) | 205 |
| **EconLit (Ebscohost)** | TI(“dementia” OR “alzheimer*” OR “cognitive impairment*” OR “cognitive dysfunction*” OR “cognitive disorder*” OR “cognitive decline*”) OR AB(“dementia” OR “alzheimer*” OR “cognitive impairment*” OR “cognitive dysfunction*” OR “cognitive disorder*” OR “cognitive decline*”))  **AND**  (TI(“Intervention*” OR “Program*” OR “Framework*” OR “Model*” OR “Service*” OR “screening”  OR “prevention*” OR “treatment*”) OR AB(“Intervention*” OR “Program*” OR “Framework*” OR “Model*” OR “Service*” OR “screening”  OR “prevention*” OR “treatment*”))  **AND**  (TI(“cost benefit” OR “cost benefits” OR “costs benefit” OR “costs benefits” OR “cost-benefit” OR “cost-benefits” OR “costs-benefit” OR “costs-benefits” OR “economic evaluation*”) OR AB(“cost benefit” OR “cost benefits” OR “costs benefit” OR “costs benefits” OR “cost-benefit” OR “cost-benefits” OR “costs-benefit” OR “costs-benefits” OR “economic evaluation*”)) | 183 |

Supplementary Table 2. Benefit items, source of data and valuation methods for benefits

| **Benefit domains and items** | **ID** | **Measurement** | **Source of data** | **Valuation method** |
| --- | --- | --- | --- | --- |
| ***Death*** | | | | |
| 1. Prevent reduction in life years | 3 | NACC data (regression estimation) | Literature | Value of statistical life |
| 1. Reduce mortality risk | 2 | NACC data (fixed effects model) | Literature | Value of statistical life |
|  | 6 | NACC data (random effects model) | Literature | Value of statistical life |
| ***Quality of life*** | | | | |
| 1. Increase QALY/Prevent QALY reduction | 1 | GDS index (one-way fixed effects model) | Literature | Value of statistical life |
|  | 2 | Clinical Dementia Rating Scale(two-way fixed effects model) | Literature | Value of statistical life |
|  | 3 | GDS index (fixed effects model) | Literature | Value of statistical life |
|  | 6 | Clinical Dementia Rating Scale (two-way fixed effects model) | Literature | Value of statistical life |
| ***Mental Health*** | | | | |
| 1. Increase well-being/mood | 8 | DEMQOL total score | HACT social value bank | Financial proxy (Value for “good overall health – age 50+”) |
| 1. Increase mental stimulation | 9 | Change in study survey (non-validated technique) | Health Innovation Network | Financial proxy (Cost of providing day care centre services) |
| ***Psychosocial*** | | | | |
| 1. Reduced fear of falling | 7 | Falls efficacy scale – International | HACT Social value bank | Financial proxy (Value for “high confidence”) |
| 1. Increase social connection and sense of belonging/Reduce social isolation and loneliness | 7 | EQ5D-3L | HACT Social value bank | Financial proxy (Value for “sense of belonging”) |
|  | 8 | Change in study survey (non-validated technique) | HACT Social Value Bank | Financial proxy (Value for “feel belonging to neighbourhood – age 50+”) |
|  | 9 | Change in study survey (non-validated technique) | Health Innovation Network | Financial proxy (Average unit cost of treating someone with depression (NHS)) |
| 1. Increase in confidence/self-esteem | 8 | DEMQOL question 5 | HACT Social Value bank | Financial proxy (Value for “high confidence – age 50+”) |
| 1. Increased feeling of control over their life/personal environment | 8 | DEMQOL question 13 | HACT Social Value bank | Financial proxy (Value for “feel in control of life – age 50+”) |
| 1. Increase engagement with art | 8 | Change in study survey (non-validated technique) | HACT Social Value bank | Financial proxy (Value for “hobbies – age 50+”) |
| 1. Increase sense of purpose | 9 | Qualitative interviews | Not valued | Not valued |
| 1. Increase of the sense of personhood and identity | 9 | Qualitative interviews | Not valued | Not valued |
| 1. Increase a sense of trust, belonging, and social wellbeing in the community | 9 | Qualitative interviews | Not valued | Not valued |
| ***Function*** |  |  |  |  |
| 1. Increase physical activity | 7 | Disability Assessment for dementia scale | HACT Social Value bank | Financial proxy (Value for “frequent mild exercise”) |
|  | 8 | DEMQOL Q10 | HACT Social Value Bank | Financial proxy (Value for “frequent mild exercise – age 50+”) |
| 1. Increase physical health | 9 | Qualitative interviews | Not valued | Not valued |
|  | 10 | EQ-5D |  |  |
| 1. Improved functions of daily living | 11 | Criterion Time for Certification of Needed Long-Term-Care | Ministry of Health, Labour, and Welfare of Japan | Financial proxy (Tarif for delivering care) |
| ***Healthcare resource use*** | | | | |
| 1. Reduction in nursing home costs | 3 | Source from literature | Medicaid budget | Direct cost savings |
|  | 13 | Connecticut Community Care | Connecticut Community Care | Direct cost saving |
| 1. Reduction in prescription drugs | 15 | Cohort study (difference-in-differences method) | Alberta Health Drug Benefit list | Market pricing-cost saving/Cost avoidance |
|  | 13 | Connecticut Community Care | Connecticut Community Care | Direct cost saving |
| 1. Reduction in inpatient hospital days | 7 | Client Service Receipt Inventory form | Literature | Direct cost savings |
|  | 13 | Connecticut Community Care | Connecticut Community Care | Direct cost saving |
| 1. Reduction in inpatient services usage | 15 | Cohort study (difference-in-differences method) | Alberta Health Services admin database | Market pricing-cost saving/Cost avoidance |
| 1. Reduction in outpatient ER visits | 7 | Client Service Receipt Inventory form | Literature | Direct cost savings |
|  | 10 | Caregiver reported | Independent Hospital Pricing Authority | Shadow price |
|  | 13 | Connecticut Community Care | Connecticut Community Care | Direct cost saving |
|  | 15 | Cohort study (difference-in-differences method) | Alberta Health Services admin database | Market pricing-cost saving/Cost avoidance |
| 1. Reduction in GP visits | 7 | Client Service Receipt Inventory form | Literature | Direct cost savings |
|  | 10 | Caregiver reported | Medicare billing code | Market price |
|  | 12 | Decision-analytic modelling | Ministry of health billing codes | Direct cost savings |
|  | 15 | Cohort study (difference-in-differences method) | Alberta Health Services practitioner database | Market pricing-cost saving/Cost avoidance |
| 1. Reduction in outpatient clinic visits | 13 | Connecticut Community Care | Connecticut Community Care | Direct cost saving |
|  | 15 | Cohort study (difference-in-differences method) | Alberta Health Services admin database | Market pricing-cost saving/Cost avoidance |
|  | 7 | Client Service Receipt Inventory form | Literature | Direct cost savings |
| 1. Reduction in caregivers’ time | 5 | Not reported | Literature | Willingness to pay to receive nursing home services (WTP) |
|  | 10 | Change in study survey (non-validated technique) | Australian Bureau of Statistics | Opportunity cost using median weekly wage (time value approach) |
| 1. Reduction in nurse time | 13 | Connecticut Community Care | Connecticut Community Care | Direct cost saving |
|  | 7 | Client Service Receipt Inventory form | Literature | Direct cost savings |
|  | 10 | Caregiver reported | Medicare billing code | Market price |
| 1. Reduction in PT/OT care | 7 | Client Service Receipt Inventory form | Literature | Direct cost savings |
|  | 10 | Caregiver reported | Medicare billing code | Shadow price |
| 1. Reduction of respite care | 10 | Caregiver reported | Caregiver reported | Shadow price |
|  | 13 | Connecticut Community Care | Connecticut Community Care | Direct cost saving |
| 1. Reduction in visits to geriatricians | 10 | Caregiver reported | Medicare billing code | Shadow price |
|  | 12 | Decision-analytic modelling | Ministry of health billing codes | Direct cost savings |
| 1. Reduction in visits to neurologist | 10 | Caregiver reported | Medicare billing code | Shadow price |
|  | 12 | Decision-analytic modelling | Ministry of health billing codes | Direct cost savings |
| 1. Reduction in visits to psychiatrist | 10 | Caregiver reported | Medicare billing code | Shadow price |
| 1. Reduction in imaging | 10 | Caregiver reported | Medicare billing code | Shadow price |
|  | 12 | Decision-analytic modelling | Ministry of health billing codes | Direct cost savings |
| 1. Reduction in lab services | 10 | Caregiver reported | Medicare billing code | Shadow price |
|  | 12 | Decision-analytic modelling | Ministry of health billing codes | Direct cost savings |
| ***Benefit to caregivers and friends*** | | | | |
| 1. Change in attitude toward people living with dementia | 8 | Change in study survey (non-validated technique) | HACT Social Value bank | Financial proxy (Value for “general training for the job”) |
| 1. Increase engagement with art | 8 | Change in study survey (non-validated technique) | HACT Social Value bank | Financial proxy (Value for “hobbies – any age”) |
| 1. Increase social support network | 8 | Change in study survey (non-validated technique) | HACT Social Value bank | Financial proxy (Value for “feel belonging to neighbourhood – any age”) |
|  | 9 | Qualitative interviews | Not valued | Not valued |
| 1. Reduce feelings of stress and burden of care | 7 | Carer strain index | HACT Social Value bank | Financial proxy (Value for “able to rely on family”) |
|  | 9 | Change in study survey (non-validated technique) | Health Innovation Network | Financial Proxy (Average cost of mental health services per individual per year) |
| 1. Change in quality of life | 10 | Change in study survey (non-validated technique) | Sourced from literature | Willingness to Pay for a QALY |
| 1. Increase well-being of caregivers | 9 | Qualitative interviews | Not valued | Not valued |
| ***Benefits for healthcare professionals*** | | | | |
| 1. Increase engagement in the community | 8 | Change in study survey (non-validated technique) | HACT Social Value bank | Financial proxy (Value for “feel belonging to neighbourhood – any age”) |
| 1. Professional development opportunity | 8 | Change in study survey (non-validated technique) | HACT Social Value bank | Financial proxy (Value for “employment training – any age”) |
| 1. Change in attitude toward people living with dementia | 8 | Change in study survey (non-validated technique) | HACT Social Value bank | Financial proxy (Value for “general training for the job”) |
| 1. Increase engagement with art | 8 | Change in study survey (non-validated technique) | HACT Social Value bank | Financial proxy (Value for “hobbies – any age”) |
| ***Wider community and society*** | | | | |
| 1. Increased knowledge in dementia (volunteers) | 9 | Change in study survey (non-validated technique) | Health Innovation Network | Financial Proxy (Cost of dementia awareness course) |
|  | 14 | Kids Insight into Dementia Survey | Not valued | Not valued |
| 1. Increased transferable skills (volunteers) | 9 | Qualitative interviews | Not valued | Not valued |
| 1. Feeling part of a community (volunteers) | 9 | Qualitative interviews | Not valued | Not valued |
| 1. Increased sense of well-being through engagement and fulfilment for volunteers | 9 | Change in study survey (non-validated technique) | Health Innovation Network | Financial proxy (Value of job satisfaction) |
| 1. Improved attitudes toward dementia, including more empathy (volunteers) | 14 | Kids Insight into Dementia Survey | Not valued | Not valued |
|  |  |  |  |  |
| 1. Improved community awareness (volunteers) | 14 | Qualitative interviews | Not valued | Not valued |
| 1. Social value of successful persecution of "serious financial offence or serious non-financial offense" | 4 | NACC data (regression estimation) | Literature | Willingness to pay for the persecution of offence |
